# Oral Contraceptive Use Is Associated With an Increased Risk of Venous Thromboembolism After Lower-Extremity but Not Upper-Extremity Arthroscopic Sports Procedures

**DOI:** 10.7759/cureus.111252

**Published:** 2026-06-21

**Authors:** Ronak J Mahatme, Shawn A Moore, Nishanth Muthusamy, Esha Reddy, Samuel K Gerak, Anish Gangavaram, Brian M Grawe

**Affiliations:** 1 Department of Orthopaedic Surgery, University of Cincinnati College of Medicine, Cincinnati, USA; 2 Department of Orthopaedic Surgery, Cleveland Clinic Foundation, Cleveland, USA; 3 Division of Sports Medicine, Department of Orthopaedic Surgery, University of Cincinnati College of Medicine, Cincinnati, USA

**Keywords:** arthroscopy, deep vein thrombosis, oral contraceptives, pulmonary embolism, venous thromboembolism

## Abstract

Background: Oral contraceptive pills (OCPs) are widely used among premenopausal women and have been associated with an increased risk of venous thromboembolism (VTE) due to the prothrombotic effects of estrogen. Although arthroscopic procedures generally carry low postoperative VTE risk, it remains unclear whether OCP use differentially influences thromboembolic complications following upper- and lower-extremity arthroscopic procedures.

Methods: This study used the TriNetX Research Network to query women aged 14-40 who underwent arthroscopic shoulder/elbow or hip/knee/ankle procedures between 2010 and 2025. Patients prescribed OCPs within six months preoperatively to three months postoperatively were compared with those not prescribed OCPs. Exclusion criteria included thrombophilia, prior VTE, hormone replacement therapy, pregnancy, and trauma. Propensity score matching was performed in a 1:1 ratio on demographic and comorbidity variables. Outcomes assessed within 90 days postoperatively included VTE, deep vein thrombosis (DVT), pulmonary embolism (PE), readmission, emergency department (ED) visits, and infection.

Results: After propensity score matching, 1,217 patients were included in each upper-extremity cohort and 6,438 patients in each lower-extremity cohort. In the upper-extremity group, OCP use was not associated with an increased risk of VTE (RR: 1.200; 95% CI: 0.520-2.767; p = 0.830), DVT (RR: 1.000; 95% CI: 0.418-2.394; p = 1.000), PE, readmission, ED visits, or infection. In the lower-extremity group, OCP users had a significantly higher risk of VTE (RR: 1.756; 95% CI: 1.199-2.573; p = 0.005) and DVT (RR: 1.714; 95% CI: 1.132-2.597; p = 0.013), but no differences in PE (RR: 1.900; 95% CI: 0.884-4.083; p = 0.137), readmission, ED visits, or infection.

Conclusion: In this retrospective propensity-matched database study, OCP use was associated with an increased risk of postoperative VTE and DVT following lower-extremity arthroscopic procedures, but not upper-extremity procedures. These findings should be interpreted in the context of procedural heterogeneity and unavailable perioperative factors, including thromboprophylaxis and postoperative mobilization. Future procedure-specific studies are warranted to better define individualized perioperative VTE risk.

## Introduction

The use of long-acting reversible contraception (LARC) has increased in the United States; however, the oral contraceptive pill (OCP) remains a common and effective form of contraception among premenopausal women [1,2]. Many OCP formulations contain estrogen in combination with progestins to prevent pregnancy [3]. Estrogen influences numerous physiologic processes, ranging from molecular signaling pathways to systemic effects across multiple organ systems [4,5]. Despite its clinical utility, supplemental estrogen was recognized early to be associated with an increased risk of thromboembolic events [6-8]. 

Orthopedic surgery is itself a relevant risk factor for venous thromboembolism (VTE), particularly deep vein thrombosis (DVT) [9,10]. Much of the existing research has focused on orthopedic trauma [11,12], where surgical invasiveness, injury severity, and prolonged immobility collectively heighten VTE risk. In contrast, elective orthopedic procedures, such as those performed in sports medicine, generally carry lower thrombotic risk, though not negligible [9]. When combined with the prothrombotic effects of estrogen-containing OCPs, elective sports procedures may pose an additive or synergistic risk of VTE. 

Anterior cruciate ligament (ACL) reconstruction has been one focal point of recent research in this area [13]. Traven et al. reported that OCP use was associated with an increased risk of DVT following knee arthroscopy, underscoring the clinical relevance of OCP-related thrombotic risk in sports procedures [14]. However, the extent to which OCPs contribute to VTE across a broader range of elective orthopedic sports procedures, and whether this risk differs between upper and lower extremity surgeries, remains poorly understood. 

The goal of this study is to use a national database to further investigate the important question of OCPs’ relationship to thrombosis after elective orthopedic sports procedures. We hypothesize that OCP use is associated with an increased risk of postoperative VTE and that the risk is greater in lower-extremity procedures compared with upper-extremity procedures, reflecting potential differences in postoperative mobility and venous stasis between these surgical locations.

## Materials and methods

The data used in this retrospective cohort study were collected from the TriNetX Research Network [15], which provided access to electronic medical records from over 150 million patients across 102 healthcare organizations. TriNetX, LLC (Cambridge, MA, USA) [15] is compliant with HIPAA and applicable data privacy regulations, and all data accessed through the platform are de-identified in accordance with federal standards. Because this study used only de-identified patient records and did not involve the collection or use of individually identifiable data, this study was exempted from Institutional Review Board approval.

Patients aged 14-40 years were included to capture the post-menarche and pre-menopause population [16]. Our cohorts who underwent elective arthroscopic orthopedic sports procedures between 2010 and 2025 were identified: (1) upper-extremity (shoulder/elbow) arthroscopy with OCP prescription within six months before and three months after surgery, (2) upper-extremity arthroscopy without OCP prescription in the same window, (3) lower extremity (hip/knee/ankle) arthroscopy with OCP prescription, and (4) lower extremity arthroscopy without OCP prescription. OCP exposure was defined based on documented prescription records within six months before surgery through three months postoperatively. Prescription documentation was used as a proxy for exposure, and medication adherence could not be confirmed. Patients with polycystic ovary syndrome, amenorrhea, prior VTE, thrombophilia, coagulation disorders, malignancy, hormone replacement therapy, pregnancy within three months, recent traumatic fracture, or polytrauma were excluded. Procedure-specific CPT distributions are reported in Appendix A to provide additional context regarding cohort composition.

Propensity score matching was performed using a 1:1 greedy nearest-neighbor algorithm without replacement. A caliper of 0.1 pooled standard deviations was used to improve covariate balance between cohorts. Propensity scores were estimated using multivariable logistic regression, including age, sex, body mass index (BMI), diabetes, hypertension, hyperlipidemia, chronic kidney disease, tobacco use, cardiovascular comorbidities, and autoimmune disease. Standardized mean differences (SMDs) <0.10 were considered indicative of acceptable balance. Only patients with complete data for matching variables were included, and no imputation was performed. Following matching, each upper-extremity cohort included 1,217 patients (Table 1), and each lower-extremity cohort included 6,438 patients (Table 2). 

**Table 1:** Propensity score matching for shoulder/elbow arthroscopic orthopedic procedures. *Event counts are less than 11 and rounded due to TriNetX limitations in reporting small event count data. BMI, body mass index; OCP, oral contraceptive pill; SD, standard deviation; SMD, standardized mean difference.

Variable	Before matching OCP (n=1,222)	Before matching control (n=250,507)	SMD before	After matching OCP (n=1,217)	After matching control (n=1,217)	SMD after
Age at index, mean ± SD	30.5 ± 12.0	50.1 ± 16.0	1.383	30.5 ± 12.0	30.5 ± 12.1	0.002
Female sex, n (%)	1,154 (94.4)	84,476 (33.7)	1.620	1,154 (94.8)	1,159 (95.2)	0.019
Male sex, n (%)	0 (0.0)	149,664 (59.7)	1.785	0 (0.0)	0 (0.0)	-
Unknown sex, n (%)	68 (5.6)	16,367 (6.5)	-	63 (5.2)	58 (4.8)	-
BMI 30–39, n (%)	57 (4.7)	15,111 (6.0)	0.067	57 (4.7)	55 (4.5)	0.008
BMI ≥40, n (%)	25 (2.0)	5,267 (2.1)	0.007	25 (2.1)	27 (2.2)	0.011
Type 2 diabetes mellitus, n (%)	34 (2.8)	23,700 (9.5)	0.289	34 (2.8)	30 (2.5)	0.021
Tobacco use, n (%)	12 (1.0)	5,156 (2.1)	0.092	12 (1.0)	13 (1.1)	0.008
Hypertension, n (%)	89 (7.3)	60,678 (24.2)	0.493	89 (7.3)	96 (7.9)	0.022
Hyperlipidemia, n (%)	67 (5.5)	43,490 (17.4)	0.392	67 (5.5)	63 (5.2)	0.015
Chronic kidney disease, n (%)	≤10* (0.8)	4,120 (1.6)	0.078	≤10* (0.8)	≤10* (0.8)	<0.001
Acute myocardial infarction, n (%)	≤10* (0.8)	2,303 (0.9)	0.013	≤10* (0.8)	≤10* (0.8)	<0.001
Rheumatoid arthritis, n (%)	11 (0.9)	2,240 (0.9)	0.002	11 (0.9)	≤10* (0.8)	0.009
Systemic lupus erythematosus, n (%)	≤10* (0.8)	556 (0.2)	0.082	10 (0.8)	≤10* (0.8)	<0.001
Heart failure, n (%)	≤10* (0.8)	2,253 (0.9)	0.011	10 (0.8)	≤10* (0.8)	<0.001
Atrial fibrillation, n (%)	≤10* (0.8)	2,639 (1.1)	0.027	10 (0.8)	≤10* (0.8)	<0.001

**Table 2:** Propensity score matching for hip/knee/ankle arthroscopic orthopedic procedures. *Event counts are less than 11 and rounded due to TriNetX limitations in reporting small event count data. BMI, body mass index; OCP, oral contraceptive pill; SD, standard deviation; SMD, standardized mean difference.

Variable	Before matching OCP (n=6,476)	Before matching control (n=445,571)	SMD before	After matching OCP (n=6,438)	After matching control (n=6,438)	SMD after
Age at index, mean ± SD	26.7 ± 10.0	40.5 ± 17.7	0.958	26.7 ± 10.0	26.7 ± 10.1	0.003
Female sex, n (%)	6,185 (95.5)	183,996 (41.3)	1.395	6,185 (96.1)	6,183 (96.0)	0.002
Male sex, n (%)	0 (0.0)	222,549 (50.0)	1.482	0 (0.0)	0 (0.0)	-
Unknown sex, n (%)	291 (4.5)	39,026 (8.8)	-	253 (3.9)	255 (4.0)	-
BMI 30–39, n (%)	228 (3.5)	19,312 (4.3)	0.052	228 (3.5)	230 (3.6)	0.002
BMI ≥40, n (%)	81 (1.3)	7,977 (1.8)	0.051	81 (1.3)	79 (1.2)	0.003
Type 2 diabetes mellitus, n (%)	66 (1.0)	19,605 (4.4)	0.219	66 (1.0)	65 (1.0)	0.002
Tobacco use, n (%)	31 (0.5)	5,331 (1.2)	0.084	31 (0.5)	30 (0.5)	0.002
Hypertension, n (%)	227 (3.5)	64,438 (14.5)	0.410	227 (3.5)	212 (3.3)	0.013
Hyperlipidemia, n (%)	180 (2.8)	45,085 (10.1)	0.318	180 (2.8)	182 (2.8)	0.002
Chronic kidney disease, n (%)	19 (0.3)	3,690 (0.8)	0.076	19 (0.3)	15 (0.2)	0.012
Acute myocardial infarction, n (%)	≤10* (0.2)	1,998 (0.4)	0.057	≤10* (0.2)	≤10* (0.2)	<0.001
Rheumatoid arthritis, n (%)	24 (0.4)	2,366 (0.5)	0.027	24 (0.4)	28 (0.4)	0.010
Systemic lupus erythematosus, n (%)	≤10* (0.2)	693 (0.2)	0.002	≤10* (0.2)	≤10* (0.2)	<0.001
Heart failure, n (%)	≤10* (0.2)	2,091 (0.5)	0.060	≤10* (0.2)	≤10* (0.2)	<0.001
Atrial fibrillation, n (%)	≤10* (0.2)	2,770 (0.6)	0.079	≤10* (0.2)	≤10* (0.2)	<0.001

Outcomes were assessed within 90 days postoperatively and included VTE (primary outcome), DVT, pulmonary embolism (PE), hospital readmission, ED visits, and postoperative infections. Information regarding postoperative thromboprophylaxis, including aspirin use, anticoagulant administration, immobilization status, and postoperative weight-bearing restrictions, was not consistently available within TriNetX and therefore could not be incorporated into the analysis.

Patient queries, demographic factors, and outcome of interest were identified using Current Procedural Terminology (CPT; a standardized procedural coding system maintained by the American Medical Association), International Classification of Diseases, 10th Revision, Clinical Modification (ICD-10-CM; a standardized diagnostic coding system maintained by the Centers for Disease Control and Prevention), and RxNorm (a standardized medication nomenclature developed by the National Library of Medicine) codes [17-19]. All codes used are provided in Appendix A.

Risk ratios (RR) and 95% confidence intervals (CIs) were computed. Complication rates were analyzed using the TriNetX system [15]. Continuous variables were evaluated with independent t-tests, while categorical variables were assessed using the chi-squared test. Statistical significance level was set at p < 0.05.

## Results

**Figure 1:**
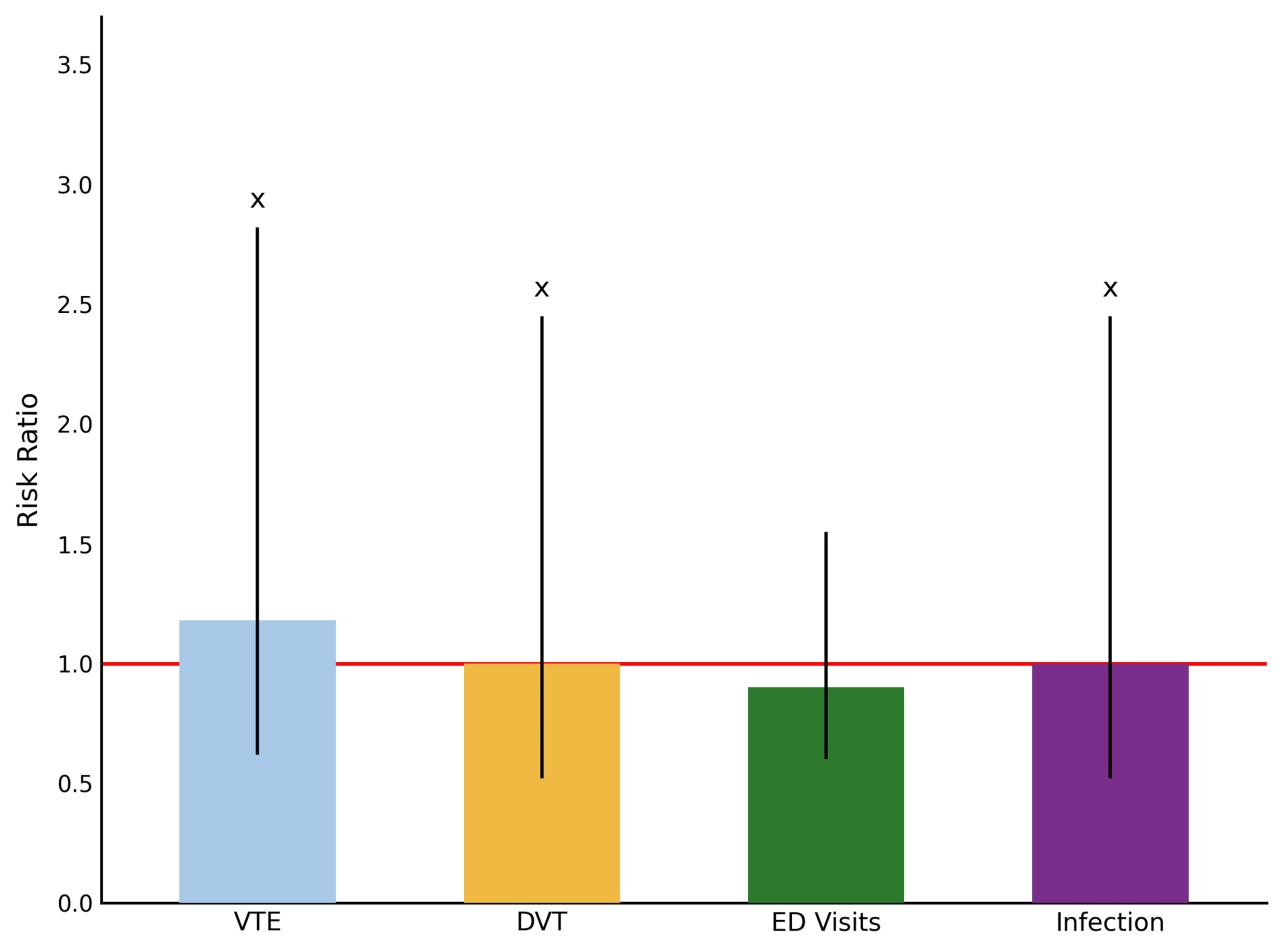
90-Day outcomes between OCP and control groups following shoulder/elbow arthroscopic orthopedic surgery. VTE: venous thromboembolism; DVT: deep vein thrombosis; ED: emergency department; OCP: oral contraceptive pill. X: ≤10 patients in at least one group.

In the matched analysis of patients undergoing shoulder and elbow arthroscopic procedures, OCP use was not associated with an increased risk of postoperative VTE, DVT, or PE. Within 90 days, no statistically significant difference in VTE incidence was observed between OCP users and controls (RR: 1.200; 95% CI: 0.520-2.767; p = 0.830). Similarly, 90-day DVT rates (RR: 1.000; 95% CI: 0.418-2.394; p = 1.000) were not statistically significant between OCP users and controls. Additionally, rates of ED visits (RR: 0.943; 95% CI: 0.590-1.507; p = 0.902) and postoperative infections (RR: 1.000; 95% CI: 0.418-2.394; p = 1.000) were not significantly different. No readmissions were observed in the OCP group, and no PEs were observed in the control group, precluding statistical comparison (Table 3, Figure 1). 

**Table 3:** 90-Day outcomes between OCP and control groups following shoulder/elbow arthroscopic orthopedic surgery. ^*^Event counts are less than 11 and rounded due to TriNetX limitations in reporting small event count data. ^†^p-values may not accurately represent significance as calculations are done with rounded patient populations. VTE: venous thromboembolism; DVT: deep vein thrombosis; PE: pulmonary embolism; ED: emergency department; OCP: oral contraceptive pill.

Outcome	OCP events (%)	Control events (%)	Total patients per cohort	Risk ratio	95% CI	p-value
VTE	12 (1.0)	≤10* (0.8)	1,217	1.200	0.520–2.767	0.830^†^
DVT	≤10* (0.8)	≤10* (0.8)	1,217	1.000	0.418–2.394	1.000^†^
PE	≤10* (0.8)	0 (0.0)	1,217	-	-	-
Readmission	0 (0.0)	≤10* (0.8)	1,217	-	-	-
ED visits	33 (2.7)	35 (2.9)	1,217	0.943	0.590–1.507	0.902
Infection	≤10* (0.8)	≤10* (0.8)	1,217	1.000	0.418–2.394	1.000^†^

**Figure 2:**
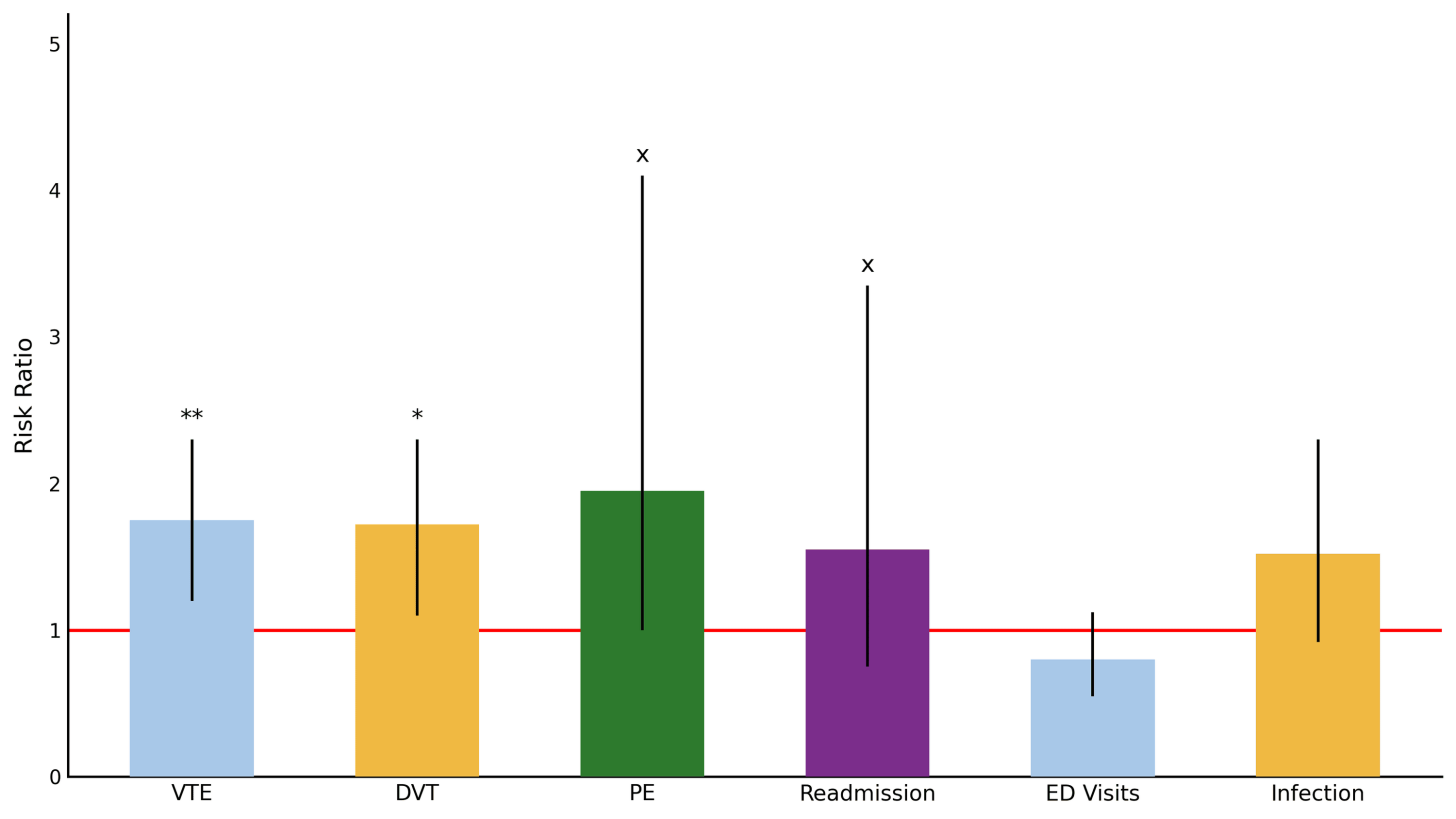
90-Day outcomes between OCP and control groups following hip/knee/ankle arthroscopic orthopedic surgery. VTE: venous thromboembolism; DVT: deep vein thrombosis; ED: emergency department; OCP: oral contraceptive pill. X: ≤10 patients in at least one group. *p < 0.05; **p < 0.01.

In contrast, among patients undergoing lower extremity arthroscopic procedures, OCP use was associated with a significantly higher risk of postoperative VTE. At 90 days, VTE occurred in 1.1% of OCP users compared with 0.6% of controls, corresponding to a 76% relative increase in risk (RR: 1.756; 95% CI: 1.199-2.573; p = 0.005). When VTE events were further stratified, OCP use was associated with increased risk of DVT (RR: 1.714; 95% CI: 1.132-2.597; p = 0.013), but not PE (RR: 1.900; 95% CI: 0.884-4.083; p = 0.137). Furthermore, OCP use was not associated with 90-day readmission (RR: 1.500; 95% CI: 0.674-3.336; p = 0.423), ED visits (RR: 0.830; 95% CI: 0.688-1.003; p = 0.060), or postoperative infections (RR: 1.440; 95% CI: 0.865-2.396; p = 0.199) (Table 4, Figure 2). 

**Table 4:** 90-Day outcomes between OCP and control groups following hip/knee/ankle arthroscopic orthopedic surgery. VTE: venous thromboembolism; DVT: deep vein thrombosis; ED: emergency department; OCP: oral contraceptive pill. ^*^Event counts are less than 11 and rounded due to TriNetX limitations in reporting small event count data. ^†^p-values may not accurately represent significance as calculations are done with rounded patient populations.

Outcome	OCP events (%)	Control events (%)	Total patients per cohort	Risk ratio	95% CI	p-value
VTE	72 (1.1)	41 (0.6)	6,438	1.756	1.199–2.573	0.005
DVT	60 (0.9)	35 (0.5)	6,438	1.714	1.132–2.597	0.013
PE	19 (0.3)	≤10* (0.2)	6,438	1.900	0.884–4.083	0.137^†^
Readmission	15 (0.2)	≤10* (0.2)	6,438	1.500	0.674–3.336	0.423^†^
ED visits	191 (3.0)	230 (3.6)	6,438	0.830	0.688–1.003	0.060
Infection	36 (0.6)	25 (0.4)	6,438	1.440	0.865–2.396	0.199

## Discussion

In this retrospective cohort study, we found that OCP use was associated with a significantly higher risk of VTE and DVT in the 90 days following arthroscopic hip/knee/ankle procedures, but not after shoulder or elbow arthroscopy. Rates of PE, readmissions, ED utilization, and infection were similar between OCP users and controls across both upper- and lower-extremity procedures. These findings suggest an association between OCP exposure and postoperative thromboembolic risk in lower-extremity arthroscopy, although causal inference cannot be established given the observational design.

Although the absolute incidence of thromboembolic events following elective orthopedic sports procedures is low (<10%), including meniscus repair and meniscectomy [20], hip arthroscopy [21], ACL reconstruction [22], shoulder arthroscopy [23], and total shoulder arthroplasty [24], the combination of high procedural volume, widespread OCP use among premenopausal women, and the prothrombotic environment of surgery means that a meaningful number of patients are exposed to increased risk [25-27]. Our results align with prior studies showing elevated VTE rates in OCP users undergoing knee and hip arthroscopy, while shoulder arthroscopy has not demonstrated increased thromboembolic risk [14,21,28,29]. No prior work has specifically evaluated elbow arthroscopy, but our findings suggest the risk is similarly low, consistent with other upper-extremity procedures. 

The differential risk between lower- and upper-extremity procedures likely reflects several physiologic and procedural factors. Postoperative immobilization of the leg contributes to venous stasis, fulfilling Virchow’s triad for thrombosis, whereas upper-extremity procedures generally allow for early mobilization. The deep venous system of the legs also experiences greater hydrostatic pressures, further predisposing to clot formation. Additionally, lower-extremity arthroscopy often involves tourniquet use, which is an established risk factor for VTE [30]. Collectively, these factors may amplify the prothrombotic effect of exogenous estrogen in OCP users undergoing lower-extremity procedures.

Interpretation of these findings should also consider the heterogeneity of oral contraceptive formulations. Prior literature demonstrates that thromboembolic risk differs across estrogen dose and progestin generation, with some third- and fourth-generation formulations associated with greater VTE risk than earlier-generation regimens [16]. Because formulation, dose, and duration were not available within TriNetX, all OCP exposure was evaluated as a pooled category, and these findings should not be interpreted as applying equally across specific contraceptive regimens.

Notably, postoperative medical utilization, including readmissions and ED visits, was comparable between OCP users and controls, suggesting that these outcomes are primarily driven by surgical factors and comorbidities rather than OCP use. Infection risk was also comparable, indicating that OCPs do not appear to significantly alter postoperative infection risk.

Our study uses a large, multicenter database and propensity score matching to reduce measured confounding; however, several limitations should be acknowledged. As with all retrospective database studies, findings depend on accurate coding of diagnoses, procedures, prescriptions, and outcomes and remain susceptible to misclassification. OCP exposure was determined from prescription records and medication adherence; formulation, estrogen dose, duration of use, and perioperative discontinuation could not be verified. Importantly, postoperative thromboprophylaxis practices, immobilization status, weight-bearing restrictions, rehabilitation protocols, and procedure-specific perioperative management were unavailable and may influence thromboembolic risk. Lower-extremity arthroscopy was analyzed as a pooled cohort despite meaningful differences between hip, knee, and ankle procedures, introducing procedural heterogeneity. Multiple outcomes were evaluated, and low event counts for select outcomes reduced statistical precision. Despite these limitations, this study provides useful observational evidence from a large propensity-matched multicenter cohort. The consistent association between OCP use and increased VTE risk within lower-extremity procedures, coupled with the absence of a similar finding in upper-extremity arthroscopy, supports continued investigation into procedure-specific perioperative thromboembolic risk assessment among OCP users.

## Conclusions

In this study, OCP use was associated with an increased risk of VTE and DVT following lower-extremity arthroscopic procedures, but not after upper-extremity procedures. While absolute rates of VTE remain low, the combination of widespread OCP use and high procedural volume suggests that a clinically meaningful number of patients may be affected. These findings support consideration of OCP exposure as one factor during perioperative VTE risk assessment and motivate future prospective studies to guide individualized perioperative management.

## Appendices

**Table 5:** CPT, ICD-10-CM, and RxNorm codes for TrinetX queries, propensity score matching, and outcomes. OCP: oral contraceptive pill; PCOS: polycystic ovary syndrome; VTE: venous thromboembolism; DVT: deep vein thrombosis; ED: emergency department; PE: pulmonary embolism; CPT: Current Procedural Terminology; ICD-10-CM: International Classification of Diseases, 10th Revision, Clinical Modification.

Diagnosis	Codes
Shoulder/elbow surgery	1005614, 1005627, 29805, 29830
Hip/knee/ankle surgery	1005648, 1005652, 29860, 29870, 29888, 29889, 1005679, 29891, 29892
OCP use	4124, 2539031
Polytrauma	T07
Pregnancy	10
Malignancy, thrombophilia, PCOS, amenorrhea, and other blood disorders	C48-C41, C81-C96, D45, D46, D47, D55-D59, D56-D64, D65-D69, D68.5, D68.6, D78-D77, E28.2, N91.0, N91.1, Z79.81
Recent fracture	S02, S12, S22, S32, S42, S52, S62, S72, S82, S92
Propensity score inputs	Age at Index, Female, E11, E78.5, I10, I21, I48.91, I50, M06.9, M32, N18, Z68.3, Z68.4, Z72.0
ED visit	99281, 99282, 99283, 99284, 99285
Postprocedural infection	T81.4
DVT	I82, T84.86, T84.81, I80.1, I80.2, 451.83
PE	I26
VTE	I26, I82, I80, T84.86, T84.81
Hospitalization	1013699
